# A Nuclei-Based Conceptual Model of (Eco)evolutionary Dynamics in Fungal Heterokaryons

**DOI:** 10.3389/fmicb.2022.914040

**Published:** 2022-05-31

**Authors:** Milica Lakovic, Matthias C. Rillig

**Affiliations:** ^1^Institut für Biologie, Freie Universität Berlin, Berlin, Germany; ^2^Berlin-Brandenburg Institute of Advanced Biodiversity Research, Berlin, Germany

**Keywords:** filamentous fungi, multinuclearity, (eco)evolutionary dynamics, heterokaryon, conceptual model

## Abstract

Filamentous fungi are characterised by specific features, such as multinuclearity, coexistence of genetically different nuclei and nuclear movement across the mycelial network. These attributes make them an interesting, yet rather underappreciated, system for studying (eco)evolutionary dynamics. This is especially noticeable among theoretical studies, where rather few consider nuclei and their role in (eco)evolutionary dynamics. To encourage such theoretical approaches, we here provide an overview of existing research on nuclear genotype heterogeneity (NGH) and its sources, such as mutations and vegetative non-self-fusion. We then discuss the resulting intra-mycelial nuclear dynamics and the potential consequences for fitness and adaptation. Finally, we formulate a nuclei-based conceptual framework, which considers three levels of selection: a single nucleus, a subpopulation of nuclei and the mycelium. We compare this framework to other concepts, for example those that consider only the mycelium as the level of selection, and outline the benefits of our approach for studying (eco)evolutionary dynamics. Our concept should serve as a baseline for modelling approaches, such as individual-based simulations, which will contribute greatly to our understanding of multilevel selection and (eco)evolutionary dynamics in filamentous fungi.

## Introduction

The Kingdom of Fungi (Eumycota) is comprised of many organisms of different shapes and sizes, from single-celled yeast to large cord-forming basidiomycetes. Fungi are eukaryotic heterotrophs with diverse life-styles, e.g., a single yeast cell interacts differently with the environment than a mycelium of a filamentous fungus. Filamentous fungi, to which we restrict our discussion here, are characterised by indeterminate growth, hyphal tip extension and anastomosis between tips, which form mycelial network. Despite a similar body concept, there are different life-history strategies across these organisms, e.g., the life-history of a long-lived sexual, dikaryotic form differs from that of a haploid asexual microfungus. Similarly, hyphal organisation within the mycelium differs between taxa (Deacon, 2006). Some have coenocytic hyphae (e.g., Mucoromycota, Chytridiomycota, and Glomeromycota) where nuclei and organelles coexist in a shared cytoplasm. Others have septate hyphae (Ascomycota and Basidiomycota) creating compartments resembling cells. Septa can be porous, allowing movement of organelles and cytoplasm between compartments. Many fungi have closed septa, however, nuclear movement among compartments is possible, *via* opening septa (van Peer et al., 2009) or cytoplasmatic bridges (Tyrrell et al., 2020). A multinucleate state is possible in compartments created by septa, where nuclei can differ in size and numbers within and between compartments (Gao et al., 2019). Furthermore, nuclear populations can change qualitatively and quantitatively depending on the developmental phase (Shahi et al., 2015), as well as the environment (Ross et al., 1991). Such nuclear organisation is especially interesting in the context of tolerance for genetic heterogeneity within the mycelium. Research focussed on nuclei in filamentous fungi has been expanding over the past decades, and data is accumulating for intra-mycelial genetic diversity (Wyss et al., 2016), pathogenicity (Ma et al., 2010; Shahi et al., 2016), nuclear dynamics (Roper et al., 2013; Roper and Dressaire, 2019; Kokkoris et al., 2020, 2021; Cornell et al., 2022) and multilevel selection (James et al., 2008; Samils et al., 2014). However, it is still a challenge to quantify nuclear movement (especially on scales larger than few cm), interactions (e.g., competition through transcription factors), genetic variability (e.g., due to spontaneous mutations) and most importantly how all these phenomena contribute to the phenotype. To what extent *nuclear genotype heterogeneity* (NGH) is occurring in mycelia in natural environments, and at what scales nuclear dispersal is important, remain open questions, yet it is essential to address these questions, especially in the context of (eco)evolutionary dynamics. Theoretical approaches, such as *individual-based models* (IBMs) are excellent tool to tackle and explore complex systems, gain additional understanding of how such systems evolve but also provide novel insights, which could lead to hypotheses testable in the lab. However, there are very few such studies that investigate nuclear genotype heterogeneity and nuclear dynamics and the potential effects on the evolution of filamentous fungi (Gifford and Schoustra, 2013; Ma et al., 2016; Scott et al., 2019; Kokkoris et al., 2020).

In filamentous fungi that harbour (i) genetically diverse nuclei (i.e., heterokaryons) with (ii) the potential to disperse through hyphal network (coenocytic hyphae, porous septa or cytoplasmatic bridges), (iii) a certain level of division autonomy (e.g., asynchronous mitosis), and (iv) totipotency (any nucleus can reproduce into the mycelium or in a spore), we can expect selection acting on different levels of organisation. These assumptions hold for many species of filamentous fungi (Deacon, 2006) belonging to different taxa, and therefore make them interesting systems for exploration of (eco)evolutionary dynamics within a mycelium. Here, we propose a conceptual model of nuclear organisation in filamentous fungi with such life-history. This conceptualisation recognises three different levels, a nucleus, nuclear group and a mycelium. In order to derive this conceptual model, we will (i) discuss sources and consequences of nuclear genotype heterogeneity, and (ii) cooperation, conflict and fitness consequences in such systems. We will then be proposing a metapopulation-like concept, suitable for individual-based modelling of (eco)evolutionary dynamics in filamentous fungi.

## Nuclear Genotype Heterogeneity and Dynamics

A fundamental necessity for deriving our conceptual model is the existence of genetic diversity among nuclei within a mycelium. We thus first discuss two important sources of NGH in filamentous fungi. We then summarise what is known and not known about the nuclear dynamics within a mycelium, because organisation and movement of genetically different nuclei influences the phenotype and ultimately evolutionary dynamics.

### Sources and Consequences of Nuclear Genotype Heterogeneity

Nuclear genotype heterogeneity occurs, for instance, in the dikaryotic stage as a part of sexual reproduction in Basidiomycetes and Ascomycetes and can persist for a period of time before karyogamy and sexual spore formation take place. Focus of this work is, however, on NGH that arises independently of sexual reproduction. Sources of such heterogeneity are somatic mutations and *vegetative non-self-fusion* (VNSF). VNSF is the result of anastomosis between hyphae belonging to genetically distinct mycelia. This can lead to nuclear exchange and heterokaryon formation (Croll et al., 2009), as well as further processes of horizontal gene transfer (Sanders, 2006), haploidisation and mitotic recombination (Schoustra et al., 2009), as a part of parasexual cycle (Pontecorvo, 1956).

#### Somatic Mutations

Vegetative mycelium grows indeterminately through hyphal tip extension and mitotic divisions of totipotent nuclei (Andrews, 1995). Somatic mutation rates, differ between species, and potentially lead to NGH within the mycelium. For example, 2–3% of stock cultures of *Neurospora crassa* and *Neurospora intermedia* become heterokaryotic, i.e., contain nuclei of different genotypes, due to spontaneous mutations (Adhvaryu and Maheshwari, 2000). Point mutations can lead to heterokaryosis where both wild type and fungicide resistant nuclei coexist (Miao et al., 2021). Interestingly, the rates at which mutations occur in some fungi have been observed to be environment-dependent (Lamb et al., 2008), e.g., increase with higher temperatures (Habig et al., 2021) and freezing (Stoycheva et al., 2007). Higher mutation rates could be just a by-product of stress-induced malfunctions or of adaptive significance. In the latter case, higher mutation rates would increase standing genetic variation for selection to act upon and it has been shown that moderate increases in mutation rates are beneficial for adaptation to novel environments (Sprouffske et al., 2018).

#### Vegetative Non-self-Fusion

Another source of genetic variation is through VNSF which can lead to heterokaryon formation and/or horizontal gene exchange (Ma et al., 2010; Shahi et al., 2016). Naturally occurring heterokaryons of many species have been isolated, e.g., *Cercospora musae* (Calpouzos, 1954), *Aspergillus nidulans* (Jinks, 1952), *Heterobasidion annosum* (Johannesson and Stenlid, 2004), *Cryphonectria parasitica* (Milgroom et al., 2009), and *Epichloë* species (Shoji et al., 2015). It was previously thought that heterokaryon formation is rare in nature due to *heterokaryon incompatibility* (HI), i.e., regulated cell death occurring at the place of fusion between the two hyphae of incompatible mycelia (Glass and Dementhon, 2006; Daskalov et al., 2017; Gonçalves et al., 2020; Rico-Ramírez et al., 2022). Whilst this is not always the case (Heller et al., 2018; Daskalov et al., 2019), evidence is accumulating that in spore germination phase, HI mechanisms may be relaxed or absent under different environmental conditions (Roca et al., 2005; Ishikawa et al., 2012; Mehta and Baghela, 2021; Vangalis et al., 2021). Fusion of two mature colonies of incompatible strains of *Colletotrichum lindemuthianum* induces cell death, while *conidial anastomosis tube* (CAT) fusion generates a stable mycelium, capable of producing uninucleate conidia phenotypically different from the parents (Ishikawa et al., 2012). CAT fusion occurs between incompatible strains of *Fusarium oxysporum* under stressful conditions, with parts of chromosomes bearing pathogenicity genes being exchanged between distinct nuclei (Shahi et al., 2016). Possibly, during early developmental stages self-recognition mechanisms can be relaxed or affected by environmental conditions, allowing formation of novel heterokaryons (Roca et al., 2005). In developed mycelia mycoviruses have been show to affect HI (Wu et al., 2017).

Heterokaryons are advantageous in variable environments because nuclear ratios can shift depending on the environment (Scott et al., 2019; Zhang et al., 2019; Cornell et al., 2022). Theoretically, if a nuclear genotype has decreased fitness, it could still be sustained within the mycelium at lower counts. If transcription is proportional to the quantity of nuclear genotype (Robbins et al., 2021), then those less abundant nuclei could have less or no influence on the phenotype, until the environment changes in their favour (Zhang et al., 2019).

### Nuclear Dynamics

Spatial and temporal organisation of nuclear divisions within the mycelium varies across species. Mitosis can be can synchronous (divisions occur at the same time), asynchronous (no discernible pattern) or parasynchronous (divisions occur as waves across neighbouring nuclei) (Pitchaimani and Maheshwari, 2003; Gladfelter, 2006). These patterns can change with developmental stage of the mycelium (Ishikawa et al., 2012). While some nuclei divide others can be subjected to autophagy (Shoji et al., 2010; Shoji and Craven, 2011; Kokkoris et al., 2020) to subsidise for resources, for example. It is not well understood how it is decided which nucleus contributes to the “next generation” or which nucleus is degraded for resources. Are these “decisions” inherent (i.e., some nuclei are genetically predetermined), imposed by nuclear autonomy (e.g., through transcription factors), environmentally induced (i.e., resource driven) spatially determined (e.g., location near the tip or in the centre of mycelium)? Answering these questions is complicated, especially in cases where a common cytoplasm is shared between nuclei. In addition, the number and size of nuclei can vary in space, (e.g., between different parts/cells of the mycelium) and in time (e.g., younger and older mycelium) (Shahi et al., 2015; Gao et al., 2019) and it is not clear what is the function, if any, of these differences.

## The Role of Nuclear Genotype Heterogeneity in Competition, Cooperation, and Fitness Consequences

Having discussed how NGH arises, and how different nuclei can be maintained or differentially multiplied, we here turn to the functional consequences of such nuclear diversity, in terms of competition and cooperation among nuclei, and the fitness consequences of such interactions for the mycelium.

### Nuclear Competition and Cooperation

Existence and the extent of NGH within the mycelium, as well as nuclear behaviour will affect the phenotype. When different genotypes share influence over the same phenotype and phenotypic effects of one genotype increase reproduction of self and reduce reproduction of the other, this is considered genetic conflict (Taylor et al., 2002; Werren, 2011). Genotypically different nuclei interacting in a common cytoplasm can affect fitness at the mycelium level (Davis, 1960). Recent research on multilevel selection in filamentous fungi reports both cooperation and competition of nuclei in different species and environments. Nuclear competition leading to non-adaptive changes has been observed in *N. crassa*, where a nuclear genotype unable to use limiting resource well, outcompetes the resource-adapted genotype, resulting in lower growth of the heterokaryon (Davis, 1960). Similarly, as a response to changing environment, competition and unbalanced nuclear ratios at the cost of mycelial performance have been shown for *Heterobasidion parviporum* (James et al., 2008). Nuclear competition occurs if coevolution between coexisting nuclei has been disrupted due to genetic changes in one of them, such as in a strain of *Neurospora tetrasperma* that acquired a recent chromosomal introgression from a closely related species (Meunier et al., 2018). Similarly, an evolutionary experiment showed that low relatedness of nuclei in *N. crassa* heterokaryon leads reduced cooperation and lower spore production due to cheater nuclei, which invest less into somatic functions and growth (Bastiaans et al., 2016). By contrast, nuclear ratios in *Rhizophagus irregularis* changed in response to the abiotic environment (Cornell et al., 2022) and a host plant shift (Angelard et al., 2010), with better adapted genotypes increasing in frequency. In addition, NGH seems to be important in fungicide resistance. Namely, shifts in resistant nuclear genotypes as response to presence of antifungals have been seen in *A. nidulans* (Zhang et al., 2019) and *Sclerotinia homoeocarpa* (Kessler et al., 2018). Indeed, a modelling study showed that NGH within fungal mycelium is evolutionary maintained because it increases survival in variable environments (Scott et al., 2019). Nuclei can cooperate to produce fitter heterokaryons not only by altering nuclear ratios but also gene expression of different nuclei (Samils et al., 2014).

### Fitness

When discussing NGH, nuclear interactions and fitness effects in fungi it is important to consider three different aspects. First, in asexual reproduction, both mycelial growth and production of spores can be assumed to contribute to fitness (Pringle and Taylor, 2002). A nucleus investing into growth can be viewed as pursuing a range expansion strategy, and securing of larger territory, while nuclei contained in spores are potential emigrants in space or/and time. Therefore, when estimating fitness, it is important to consider both components, because there might not be trade-offs and predictable relationship between mycelial growth and sporulation (Anderson et al., 2019). A second important aspect of fitness is how is NGH reflected in spores. Asexual spores can be uni- or multinucleate, a trait that differs between species (Kokkoris et al., 2020) as well as within the same mycelium (Okungbowa and Shittu, 2014). Regardless, it would be expected, in the absence of division of labour, that spore genotypes reflect NGH of the heterokaryon. However, nuclear transmission from heterokaryon to spores can be biassed toward a single nuclear genotype (Meunier et al., 2018; Robbins et al., 2021). This implies that nuclear competition extends to sporulation and can lead to decreased fitness of germlings. However, nuclei can cooperate and Ma et al. (2016) show that mycelium of *N. crassa* is comprised of subpopulations of nuclei that reproduce together and function as “reproductive units.” The third point to consider is that the distribution of NGH across the mycelium can create phenotypic heterogeneity within mycelium where selection could then act on this heterogeneity. While some studies show that nuclear ratios are evenly distributed or homogenised across the mycelium (Meunier et al., 2018; Cornell et al., 2022) there is evidence of uneven nuclear distribution as well (He et al., 2021). Homogenisation is possibly achieved *via* nuclear movement through mycelium (Roper et al., 2011). However, such observations are based on small scale Petri dish lab systems and we have no data if and how nuclei move and homogenise at larger scales. Estimates of the real sizes of fungal mycelia in natural environments, such as soils, are difficult. For instance, estimates that *Armillaria bulbosa* is the largest organism on earth (Smith et al., 1992) show just how big fungi can get, but it is not clear if such a spatial extent also encompasses physiological integration. If we assume that in a large physiologically integrated mycelium nuclear movement cannot occur effectively throughout the entire body, this would open the possibility that different genotypes and phenotypes could segregate within one mycelium, leading to differential fitness of distinct fungal parts. From the natural selection perspective, these phenotypes can be viewed as two different entities with different fitness, where a group of nuclei contributing to the better performing phenotype is favoured.

## A Nuclei-Based Conceptual Model of (Eco)Evolutionary Dynamics

As selection ultimately targets the phenotype, we consider that different levels of fungal organisation interact in an unconventional way, compared to unitary organisms, to generate those phenotypes. Phenotypic heterogeneity is common for filamentous fungi (Hewitt et al., 2016; Silar, 2019) where distinct phenotypes are observed across the mycelium (Levin et al., 2007; Op De Beeck et al., 2020; Tegelaar et al., 2020) sometimes even parts deprived of cytoplasm and nuclei (Klein and Paschke, 2004). If the communication and connectedness between such different phenotypes is weak or lacking, then they can be described as different units upon which selection acts independently. When a mycelium reaches a critical size, it may be a good strategy to disconnect communication between distant parts, because of the costs of maintaining such an integrated network. In such a situation, a subpopulation of nuclei, operating in proximity to each other (i.e., belonging to the same or neighbouring hyphae), generating a certain phenotype, is likely to be selected together. Therefore, inspired by the idea of “reproductive units” (Ma et al., 2016), we propose a conceptual model of three-level fungal organisation as spatially structured populations (metapopulation) of nuclei.

### Conceptual Model

**FIGURE 1:**
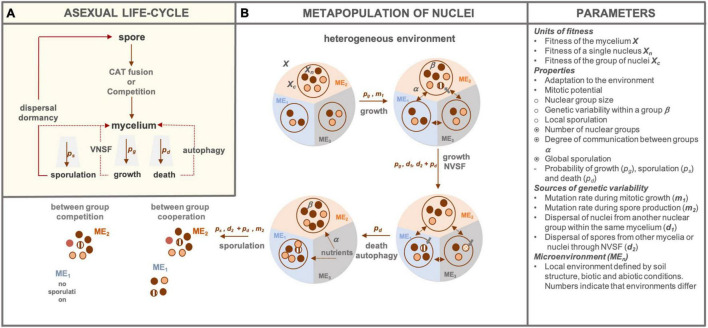
Schematic representation of the asexual fungal life-cycle and nuclear metapopulation. **(A)** We show an asexual spore which can, following germination, undergo CAT fusion or competition if other spores are present. Germlings develop into a mycelium that can die, grow and/or sporulate with probabilities *p*_*d*_, *p*_*g*_ and *p*_*s*_, respectively. Dashed lines indicate alternative events that can take place. That is, if death occurs only in parts of the mycelium, autophagy can take place, providing energy reserves to subsidise growth or sporulation of other mycelial parts. Similarly, a growing mycelium can fuse with other genetically distinct mycelia through VNSF. In a sporulation event, spores may fuse with an existing mycelial network or pass through the cycle again independently. **(B)** We show a mycelium as a metapopulation of nuclei in a heterogenous environment (ME_1_,_2_,_3_), with fitness of the mycelium, nuclear group and an individual nucleus denoted as *X*, *X*_*c*_, and *X*_*n*_, respectively. If the environmental conditions allow, the mycelium will grow through mitotic divisions of nuclei, which introduces mutant nuclei (denoted with grey bolded arrow) at a species-specific rate *m*_1_. Within-group genotype diversity, denoted as β, can be the same or different between groups depending on within-mycelium nuclear mixing, denoted by α, the connectivity parameter. Nuclear subpopulations can change quantitatively and/or qualitatively by immigration from other groups (*d*_1_) or by introduction of distinct genetic material through successful (depending on the *p*_*d*_) VNSF (*d*_2_). Similarly, older or resource-deprived parts of mycelium may undergo cellular death and autophagy, which can subsidise other mycelial parts with energy for growth or sporulation. Sporulation can occur across the mycelium if there is between-group cooperation. If there is competition between the groups, some groups may not contribute or contribute less to the spore pool. Genetic diversity may be introduced in the spore pool through successful immigration (depending on the *p*_*d*_) of spores from another mycelium (*d*_2_) or mutations and mitotic rearrangements during spore production (*m*_2_). The last panel on the right (“Parameters”) provides explanations for denotations used in the scheme and a short list of properties at every level with •, ￮, 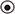 representing nuclear, group and mycelium level, respectively. Similarly, “-” represents traits that can be attributed to either of the levels. Different colours of nuclei or spores indicate genetic differences.

We use a simplified life-cycle of asexual fungus (Figure 1A) where a successfully germinated spore undergoes CAT fusion or competes with other spores. The resulting mycelium may grow, sporulate or die with a certain probability *p*_*g*_, *p*_*s*_, and *p*_*d*_, respectively. These life events may occur at different points in time but also in space. For example, cell death occurring in one section of the mycelium may lead to autophagy resulting in nutrient subsidy of other mycelial parts (Shoji and Craven, 2011). VNSF may occur if a growing hypha encounters genetically distinct mycelium and successful anastomosis results in (heterokaryotic) mycelium. Spores can either germinate and fuse with the mycelium of origin (Sbrana et al., 2011) or disperse in space and/or time until conditions for germination are met and the cycle repeats. Because filamentous fungi operate at micro, hyphal, and macro, mycelium scale, distinct parts of the fungal body may experience different microenvironmental conditions, especially in complex environments, such as soils. Consequently, as well as given the nuclear totipotency and lack of division of labour, different phenotypes may arise across a single mycelium. Such intra-mycelium phenotypic heterogeneity may lead to differences in growth, sporulation and death. As outlined earlier, a single nucleus or a mycelium have been discussed as units of selection in filamentous fungi. Considering individual nuclei is important because NGH can arise at any time during the growth phase *via* spontaneous mutations, mitotic recombination (*m*_1_) or introduction of new nuclei through VNSF or CAT fusion (*d*_2_). When the environmental conditions change, nuclear ratios change if better adapted nuclei can propagate at higher rates, which will in turn increase the fitness of the mycelium. Alternatively, cheater nuclei could have higher fitness at the nuclear level, leading to a fitness cost of the mycelium. The genetic diversity of spores, can, therefore, differ between microenvironments and these spores can cooperate (CAT fusion) or compete during germination. Adaptation through standing genetic variation is likely to lead to faster evolution (Barrett and Schluter, 2008), especially in dynamic environments such as soil, hence it is important to account for NGH explicitly. However, because selection acts on phenotypes, which cannot be produced by a single nucleus but rather a subpopulation of nuclei, we propose such subpopulations of nuclei be considered as the third level of selection. Therefore, a mycelium can be viewed as a sort of *metapopulation of nuclei* (Figure 1B) that emerges as a result of mycelial architecture and/or environmental heterogeneity.

By mycelial architecture, we refer to realised communication and transport across the network. This is the connectivity parameter (α) that represents dispersal potential between the groups. For example, when communication is not homogenous across a connected mycelium, phenotypic heterogeneity can be created even in a homogenous environment. In a given environment, the fitness of a single nucleus (*X*_*n*_) depends on its adaptation as well as quantity and the quality (β) of surrounding nuclear genotypes, e.g., even dominant gene products could be diluted in heterokaryon and therefore do not produce the dominant phenotype (Casselton and Lewis, 1967). Alternatively, considering a group with two different genotypes in which 70% of nuclei have a specific gene coding for an enzyme do degrade available substrate, the rest will also benefit from acquired resources. We assume that within a group, nuclear and nutrient mixing is maximised due to hyphal proximity, interconnectedness and growth-driven transport of elements. Therefore, at the subpopulation level growth and nuclear content is driven by the microenvironment and parts of mycelium experiencing different microenvironments will have different fitness (*X*_*c*_). However, if communication between different groups (α) is high, then nutrients or nuclei may be transported to less fit part(s), which can be both beneficial or detrimental to the mycelium. For instance, nutrient transport to subsidise growth in a resource poor subpopulation is beneficial if that part is worth preserving (e.g., securing larger territory until new resources appear); however, sometimes a better strategy would be to keep growing or sporulating in one part, disconnecting the rest of the mycelium or even degrading it through autophagy to gain more nutrients (e.g., when the expectation of new resource inflow is low). Therefore, the parameter that defines area and speed of transport (α), is an aspect of species-specific life history that evolved in response to a specific environment. Such traits will differ between species living in relatively nutrient stable environments (e.g., wood decaying fungi) and those living in patches with ephemeral resources (e.g., soil microfungi). Subpopulation size, or the extent of the phenotype under selection, is a dynamic property and depends on both lower and higher-level interactions. That is, the fitness of individual nuclear genotypes depends on their own adaptation to the given microenvironment and on what other nuclei in the group are doing. If the group is performing well, it can bring more resources for growth and increase in size. At the level of the mycelium, network architecture determines how well nuclei and resources mix between different parts, representing a top-down regulator of group size. Network connectivity depends on self-anastomosis rates, which can differ between species and developmental stages, making self-fusion another life-history trait worth considering. Subpopulations may, as well, go extinct in case of competition, predation or autophagy (Shoji et al., 2010; Shoji and Craven, 2011; Kokkoris et al., 2020).

## Conclusion

We have highlighted the benefits of a nuclei-centric approach for theoretical exploration of filamentous fungi that possess certain, commonly observed life-histories. Such bottom-up approaches allow exploration and identification of candidate mechanisms for understanding emergent behaviour at the mycelial level. Our holistic depiction of fungal organisation as spatially structured populations of nuclei could play a central role in improving our understanding of adaptation as well as (eco)evolutionary dynamics in filamentous fungi. How and why is NGH maintained? What drives competition and cooperation in multinucleated systems with NGH? Is there an upper limit for NGH? What are the conditions for having spatially homogenous versus heterogenous NGH? How does all this relate to the phenotype that is under the selection? And finally, this conceptualisation is ideally suited to providing insights into multilevel selection in filamentous fungi. We suggest that it should be a research priority to combine theoretical and experimental nuclei-oriented approaches, since this has the potential to address these and many other key questions in fungal ecology and evolution, and also to predict responses in the face of global change.

## Data Availability Statement

The original contributions presented in the study are included in the article/supplementary material, further inquiries can be directed to the corresponding author.

## Author Contributions

ML and MR contributed to drafting and revising the manuscript. ML designed the figure. Both authors are in agreement with the submitted version of the manuscript.

## Conflict of Interest

The authors declare that the research was conducted in the absence of any commercial or financial relationships that could be construed as a potential conflict of interest.

## Publisher’s Note

All claims expressed in this article are solely those of the authors and do not necessarily represent those of their affiliated organizations, or those of the publisher, the editors and the reviewers. Any product that may be evaluated in this article, or claim that may be made by its manufacturer, is not guaranteed or endorsed by the publisher.
